# A new nanoscale transdermal drug delivery system: oil body-linked oleosin-hEGF improves skin regeneration to accelerate wound healing

**DOI:** 10.1186/s12951-018-0387-5

**Published:** 2018-08-30

**Authors:** Weidong Qiang, Tingting Zhou, Xinxin Lan, Xiaomei Zhang, Yongxin Guo, Muhammad Noman, Linna Du, Jie Zheng, Wenqing Li, Haoyang Li, Yubin Lu, Hongyu Wang, Lili Guan, Linbo Zhang, Xiaokun Li, Jing Yang, Haiyan Li

**Affiliations:** 1 College of Life Science, Engineering Research Center of the Chinese Ministry of Education for Bioreactor and Pharmaceutical Development, Jilin Agricultural University, Changchun, 130118 China; 2 Jilin KingMed Center for Clinical Laboratory Co., Ltd, Changchun, 130000 China

**Keywords:** Epidermal growth factor, Immunohistochemistry, Oil body, Skin regeneration, Wound healing

## Abstract

**Background:**

Epidermal growth factor (EGF) can promote cell proliferation as well as migration, which is feasible in tissue wound healing. Oil bodies have been exploited as an important platform to produce exogenous proteins. The exogenous proteins were expressed in oil bodies from plant seeds. The process can reduce purification steps, thereby significantly reducing the purification cost. Mostly, the diameter of oil body particle ranges between 1.0 and 1.5 µm in the safflower seeds, however, it reduces to 700–1000 nm in the transgenic safflower seeds. The significant reduction of particle size in transgenic seeds is extremely beneficial to skin absorption.

**Results:**

The diameter of oil body in the transgenic safflower seeds was recorded in the range of 700–1000 nm. The smaller particle size improved their skin absorption. The expression level of oleosin-hEGF-hEGF in T3 transgenic seeds was highest at 69.32 mg/g of seeds. The oil body expressing oleosin-hEGF-hEGF had significant proliferative activity on NIH/3T3 cells and improved skin regeneration thereby accelerating wound healing in rats. The wound coverage rate exceeded 98% after treatment for 14 days with oil body expressing oleosin-hEGF-hEGF, while the saline without EGF group and wild type oil body group both showed less than 80%. The neonatal fibroblast and collagen were found to be increased in the safflower oil body expressing oleosin-hEGF-hEGF treatment group. TGF-β1, bFGF and VEGF were noted as important growth factors in the repair of cutaneous wounds. Their expression level increased after 4 and 7 day treatment, but decreased after 14 days. Therefore, it can promote skin regeneration to accelerate wounds healing.

**Conclusions:**

The expression of oleosin-hEGF-hEGF in T3 transgenic seeds was 80.43 ng/μL oil body. It had significant proliferative activity on NIH/3T3 cells and improved skin regeneration to accelerate wound healing in rats. The expression process of TGF-β1, bFGF and VEGF increased at first and then gradually declined.

**Electronic supplementary material:**

The online version of this article (10.1186/s12951-018-0387-5) contains supplementary material, which is available to authorized users.

## Background

hEGF is a small molecule polypeptide that forms three pairs of disulfide bonds during protein folding, which is necessary for its biological activity [1]. hEGF promotes skin burns, traumatic skin ulcers and diabetes-induced body ulcers [2–4]. hEGF is able to bind to EGFR on the cell membrane near the wound [5], activates the protein kinase, and thus accelerates the synthesis of DNA, RNA and protein. Ultimately they promote the cells near wound to speed up the mitosis [6], making the wound to heal effectively [7].

Wound healing is a complicated process involving a series of successive and folded stages, such as clotting, inflammation, proliferation, and remodeling [8]. In this process, not only the process of cell proliferation, differentiation, migration, apoptosis and disappearance occur [9], but also a series of different types of cells, structural proteins, growth factors and protein kinases and other network-forming interactions result [10]. It is evident that growth factors can regulate the various stages of wound repair [11]. These growth factors include fibroblast growth factor(FGF), vascular endothelial growth factor (VEGF), hepatocyte growth factor (HGF), epidermal growth factor (EGF) and transforming growth factor (TGF) as well as others [12]. Among them, TGF-β1, bFGF and VEGF are the main factors involved in the later inflammation of wound and repair of a proliferative period [13]. VEGF promotes the proliferation of vascular endothelial cells and the formation of capillaries in granulation tissue. Inducing the neutrophils and macrophages to accumulate into wounds, TGF-β1 promotes fibroblast differentiation and cell matrix production. bFGF is mainly related to the synthesis of collagen fibers [14], promoting wound healing, scar formation, proliferation and contracture of functional cytokines.

In recent years, the research hotspot for the plant bioreactor is the use of oil body-oleosin technology [15] to produce foreign protein. The oil body could express exogenous proteins in oil crops seed, which can target exogenous proteins to the surface of oil bodies via covalent fusions with oleosin [16]. This has obvious advantages like lower production costs, more convenient storage, more stable heredity and higher output potential [17–19], compared with bacterial, fungal, insect and mammalian expression system.

Oil body is a spherical subcellular organelle with 0.5–2.5 μm diameter storing oil present inside the plant seed [20]. Oilseeds store lipids in oilbodies, which are relatively simple organelles comprising a matrix of TAG coated by a phospholipid monolayer embedded by oleosins [21]. Oleosin consists of three parts: N-terminus variable region, C-terminus variable region, and intermediate hydrophobic region [22]. The C- and N-termin are embedded on the surface of the oil body and exposed to the cytoplasm. This configuration generates resistance as well as electrostatic repulsion so oil body can exist independently [23]. It is evident that the accumulation of oleosin regulates the size of oil bodies [21]. The aberrant phenotype was reversed by reintroducing oleosin, which decreased thee size of the oil body and enhanced its stablility by the accumulation of oleosin [21].

The oil body is similar to lipidosome and widely used in biotechnology on account of the oleosin structure and its specificity. For example, it can be used as emulsifier to carry and fix the recombinant protein. It can also be used to purify and refold recombinant protein. The Sembiosys in Canada uses oil body bioreactors to express a variety of pharmaceutical proteins [24]. Presently, with the exploitation of safflower oil body as a bioreactor, we obtained oleosin-hEGF-hEGF protein. A large number of transgenic safflower oil bodies were obtained at high yield. The oleosin-hEGF-hEGF expressed in safflower seeds was calculated to be 69.32 mg**/**g of seeds. The size of the transgenic oil bodies decreased and dropped to the nanoscale level, which leading to the development of a new drug carrier that could carry hEGF.

into the skin and accelerate the treatment of skin wounds.

## Methods

### Experimental materials

The safflower seeds were purchased for Xinjiang Tacheng Honghuayuan Biotechnology Co., Ltd. and were multiplied at experimental field of Jilin Agricultural University. T3 transgenic safflower seeds were preserved at Engineering Research Center of the Chinese Ministry of Education for Bioreactor and Pharmaceutical Development. The T-DNA region of the pOTB expression contained the phaseolin promoter, oleosin gene, phaseolin terminator, 35S promoter, bar gene, and nos gene [25]. The hEGF-hEGF gene was inserted into the pOTB expression vector to construct pOTB-hEGF-hEGF recombinant plasmid. Then the recombinant plasmid was transformed into competent cells of Agrobacterium tumefaciens, and cotyledonary node was transformed by Agrobacterium-mediated method to obtain transgenic safflower plants. The T3 transgenic safflower lines T3-13, T3-14, and T3-17 with relative higher expression were identified at the molecular level.

### Oil body extraction

The safflower oil body was extracted using gradient centrifugation according to methods of Tzen et al. [26] and Jacks et al. [27]. The T3 transgenic and wild-type safflower seeds (each 20 g) were kept immersed in 100 mL of distilled water respectively for overnight at 4 °C. They were placed into 200 mL ddH_2_O, Tris–Cl (BeiJing DingGuo ChangSheng Biotechnology Co., Ltd., China), NaOH (Sinopharm Chemical Reagent Co., Ltd., China), Tricine (BeiJing DingGuo ChangSheng Biotechnology Co., Ltd., China) and PBS (Beijing solarbio science&technology Co., Ltd., China) buffer (all had pH value of 7) and ground for 3 min in a colloid mill. The mixture was filtered and the filterate was centrifuged at 12,000 g and 4 °C for 15 min. The suspension was collected, blended again and emulsified for 2 min with different extraction buffer (ddH_2_O, Tris–Cl, NaOH, Tricine and PBS buffer). Then the emulsified liquid was centrifuged at 10,000 g at 4 °C for 15 min. The recovered supernatant was resuspended with extraction buffer, emulsified for 2 min and centrifuged at 8000 g for 15 min at 4 °C [19]. Finally, the pure oil bodies were recovered and stored at 4 °C. The optimal extraction solution was selected in accordance with extraction rates of oil bodies. Thereafter, different pH (6–8.5) values of the optimal extraction solution were compared and screened. The microscopic structure of oil body was dyed and observed by nile red (Shanghai Biological Technology Development Co., Ltd., China) and the diameter of particles was detected by laser particle analyzer (PSS Nicomp380 ZLS, USA).

### Oleosin-hEGF-hEGF fusion protein analysis

The oil body was diluted with PBS to 5 μg/µL, added 1 × loading buffer and the suspension was heated at 90 °C for 10 min. The oil body suspension was analyzed by 15% polyacrylamide gels (Beijing solarbio science & technology Co., Ltd., China). One of the gels was dyed by Coomassie Brilliant Blue (CBB) (BeiJing DingGuo ChangSheng Biotechnology Co., Ltd., China) staining method and decolored for 24 h with decolorization solution. Another gel was used to transfer protein to a PVDF membrane (BeiJing DingGuo ChangSheng Biotechnology Co., Ltd., China). It was blocked overnight with 20 mL of TBST buffer (pH = 8.0) (Beijing solarbio science & technology Co., Ltd., China) including 0.05% Tween-20 (MYM Biological Technology Co., Ltd., China) and 5% nonfat dry milk (BD Difco, USA). The blocked PVDF membrane was washed 4 times (8 min each time) with TBST buffer and incubated with a rabbit anti-hEGF polyclonal antibody (1:2000, Abcam, ab9605, Lot: GR11004-41 USA) followed by the secondary antibody which is goat anti-rabbit IgG/AP antibody (alkaline phosphatase-conjugated, Promega, S3738, Lot: 00001473 46, USA) [17]. The oleosin-hEGF-hEGF protein accumulation data in safflower seeds was analyzed with Quantity One software.

### Oleosin-hEGF-hEGF proliferation assay

NIH/3T3 cell line (supplied by Wenzhou Medical University) was cultured in 25 cm^2^ culture flask having the growth medium with low-sugar DMEM (Hyclone, Thermo Fisher Scientific, USA), 50 μg/mL streptomycin (Beyotime Institute of Biotechnology, China), 100 μL/mL penicillin (Beyotime Institute of Biotechno-logy, China) and 10% (V/V) fetal bovine serum (FBS) (Thermo Fisher Scientific, USA). The 3T3 cells in logarithmic growth phase were then transferred to 96-well plate (5 × 10^4^ cell per well). The 100 μL low-sugar DMEM medium containing 0.4% FBS was added to every well and incubated in 37 °C incubator for 24 h. The 3T3 cells were allowed to incubate for 48 h after adding wild-type safflower oil body (negative control), standard bFGF protein, and safflower oil body expressed oleosin-hEGF-hEGF (initial concentration of 125 ng/mL). Afterwards, 25 μL MTT (Thermo Fisher Scientific, USA) was injected into each well and cultured for 4 h at 37 °C. Finally, the medium was out-welled, added 100 μL DMSO (Beijing solarbio science & technology Co., Ltd., China) per well and cultured for 10 min. The absorbance values were detected at 570/630 nm.

### Transdermal absorption

Twenty-four male and female ICR mice were weighed 20–22 g (Yisi experimental animal technology company, China). These mice were randomized into 3 groups (n = 6). All mice were fainted by injection 5% chloral hydrate solution. The hair of mice’s backside was shaved and depilated completely. The 40 µL of physiological saline buffer was applied on the back as a blank control group (Group A). The 20 µg EGF protein in 40 µL PBS solution was smeared as a positive control group (Group B). The 40 µL of the oil body expressed oleosin-hEGF-hEGF fusion protein was smeared as a sample group (Group C). The medication area was taken 1 cm^2^ on mice’s backside. The skin tissues were collected after transdermal absorption for 30, 60 and 90 min duration in each group respectively. The collected tissues were embedded in which paraffin sections were created. Tissue slices were blocked and then incubated with the primary (rabbit Anti-hEGF, Bioss, bs-4568R, Lot: C-A1729, China) and secondary antibodies (Goat anti-Rabbit IgG ZSGB-BIO, ZLI-9019, Lot:K176904D, China). These tissue sections were stained with DAB substrate kit (Solarbio, China) and then hematoxylin (Solarbio, China) counterstain for 1 min. All photos were recorded using an Olympus confocal microscope (Olympus, Japan) [28].

### Establishment of wound model in rats

Wistar rats were provided by the Jilin YiSi Experimental Animal Center (200–220 g). Forty male and female adult Wistar rats were randomly divided into four groups, with each group containing 10 rats. They were anesthetized with 7% chloral hydrate (Win–Win Chemical Co., Ltd), fixed on a simple console after anesthesia and then shaved their hairs on their backside. Circular wound of diameter 1.8 cm was made in the backside of the spine as the axis of symmetry with the Skin biopsy perforation machine (Acuderminc., Ft Lauderdale, FL, USA).

### Treatment and analyses of wound healing

After wounding, these rats were treated by drug and divided into four experimental groups. Group A: saline without hEGF (blank control group); Group B: wild type oil body of safflower (negative control group); Group D: safflower oil body expressed oleosin-hEGF-hEGF (sample group) (1 mg/cm^2^/d); Group C: EGF standard protein (positive control group) (1 mg/cm^2^/d). All the four groups were photographed every day of the treatment for 14 days. To calculate the wound areas and healing rate, images of the wound were taken at postoperative 0, 4, 7 and 14 days. The images were analyzed by software Image J (National Institutes of Health, USA) to obtain the corresponding wound area and healing rate. Wound healing rate = (initial wound area − final wound area)/initial wound area × 100%.

### Histological analysis and immunohistochemical staining

The skin tissues were collected and fixed in 10% neutral paraformaldehyde for 24 h. They were embedded and paraffin sections were made. A part of tissue sections were stained by hematoxylin and eosin (HE) to obtain the thickness of granulation tissue and the formation of normal dermis substructures. Another part of tissue sections were stained using FeCl3-hematoxylin solution for 6 min and ponceau S and magenta solution for 3 min. They were rinsed with weak acid solution for 2 min and stained with aniline blue for 1 min. The third part of tissue sections were put into citrate buffer and heated for 10 min for antigen recovery.

After washing, the slides were added to the bovine serum albumin solution for 20 min at room temperature and kept overnight at 4 °C. Part of the slides were added to the primary antibody overnight (anti-PCNA, BOSTER Biological Technology co. ltd, bm0105, Lot:11H05, China), goat anti-mouse IgG H & L (HRP), ZSGB-BIO, sp-9002, Lot:K176918G, China) was added dropwise at 37 °C for 20 min, and the sections were treated with DAB staining kit. Another portion of the slides was loaded with anti-CD31 antibody (Bioss, bs-0195R, Lot:AD175629, China) followed by fluorescein-conjugated goat anti-rabbit IgG(H+L secondary antibody (ZSGB-BIO, ZF-0311, Lot:65214G8, China), finally treated with DAPI (Solarbio, China) [28]. The morphological changes of neonatal skin tissues were observed under different treatment conditions.

### Transcription analysis of TGF-β1, bFGF and VEGF mRNA in neonatal skin

Wistar rats were killed by cervical vertebra sudden death on day 4, 7 and 14. The neonatal skin obtained was subjected to immunohistochemical staining test. The total RNA was then extracted by Trizol (Thermo Fisher Scientific, USA) following manufacturer’s instructions. The RNA quality was determined based on OD260/280 values by Nano Drop 2000 (Thermo Fisher Scientific, USA) while its integrity was detected by 1.2% agarose gel electrophoresis. The total RNA (1 μg) was reverse transcribed. Real-time quantitative PCR was performed on Stratagene Mx3000P thermocycler (Agilent). Gene-specific primers for the TGF-β1, bFGF, VEGF genes and candidate reference β-actin gene were designed (Additional file 1: Table S1). RT-qPCR reaction procedures were as following; pre-denaturing stage: 95 °C, 30 s; PCR reaction stage: 40 cycles of 95 °C for 5 s and 60 °C for 15 s. The fold change in relative expression level was calculated with 2^−△△CT^ method.

### Dynamic expression of TGF-β1, bFGF and VEGF proteins in neonatal skin tissue

At three time points (day 4, 7 and 14), the neonatal skin tissues of rats were collected for the immunohistochemical staining test. The total proteins were extracted from the neonatal skin tissues using the total protein extraction kit (Solarbio company, China) according to manufacturer’s instructions. The total protein solution of each group was added 50 µL 5 × loading buffer to boil for 10 min. Then, the protein samples from skin tissues of day 4, 7 and 14 were detected via western blotting. Each group of PVDF membranes were incubated by rabbit anti-bFGF, anti-TGF-β1, anti-VEGF and anti-β-actin polyclonal antibody antiserum (abcom, ab10420, Lot: GR1798 08, USA; Bioss, bs-0086R, Lot: AG07188387; Bioss, bs-4572R, Lot:AG08043197; Bioss, bs-0061R, Lot: AG07197903 China) and then the secondary antibody was incubated using goat anti-rabbit IgG antibody with alkaline phosphatase (Promega, USA). Finally, all images were visualized with AP developer (Promega, USA) for 1 min. This experiment was repeated three times.

### Statistical analysis

The data were counted by GraphPad Prism 6.01 software (Inc., La Jolla, CA, USA), For all these statistical data, *p < 0.05, **p < 0.01 and ***p < 0.001 were considered statistically significant.

## Results and discussion

### Extraction and particle size detection of oil body

**Fig. 1:**
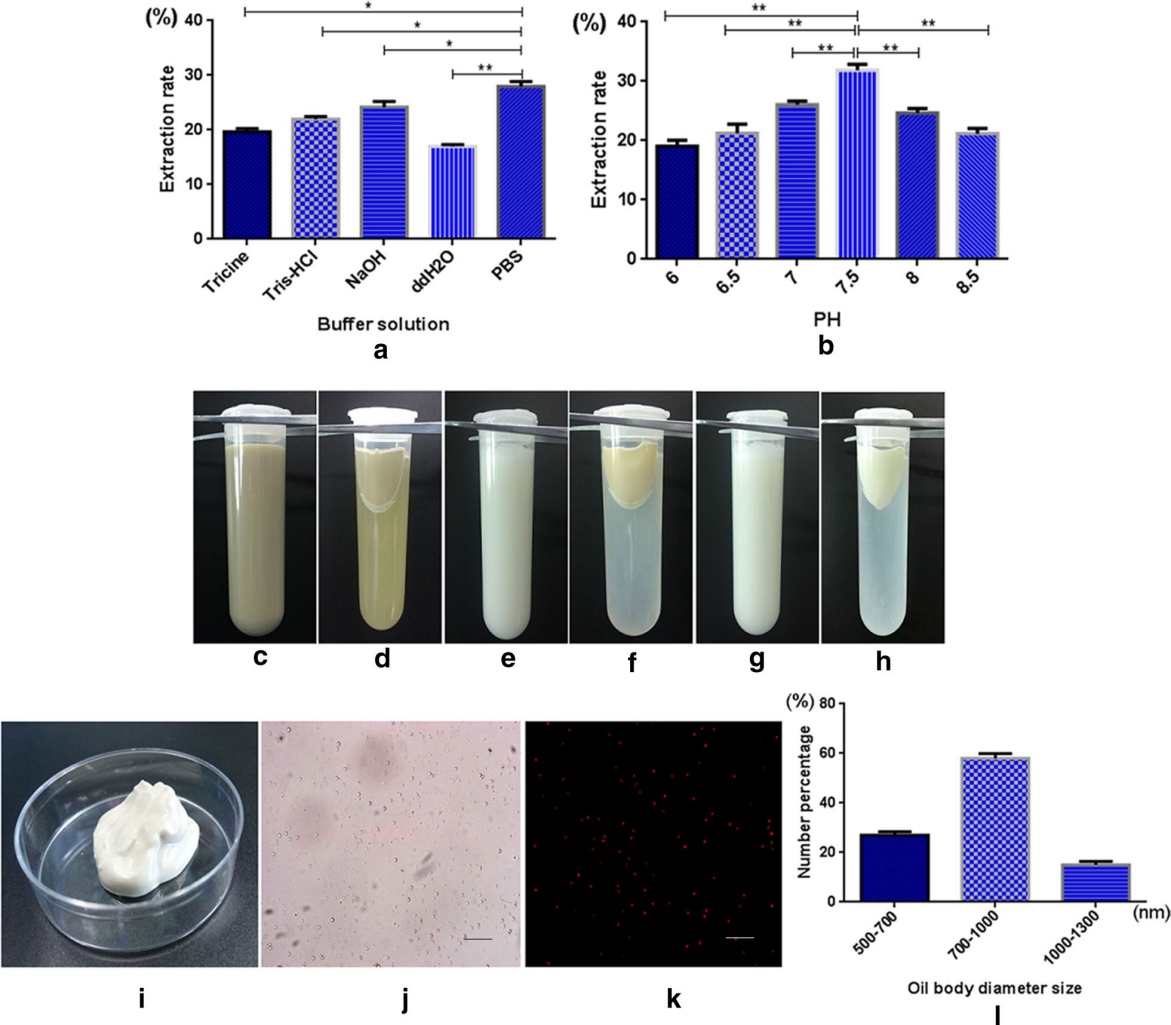
Extraction process of oil bodies and analysis of microstructure and partical size. **a** Compared with extraction rate by different extraction buffer; **b** compared with extraction rate by different pH value; **c** the mixture after ground in colloid mill; **d** the first centrifuged product; **e** the resuspending after washing for the first time; **f** the second centrifuged product; **g** the resuspending after washing for the second time; **h** the third centrifuged product; **i** the pure oil body from T3 transgenic safflower seeds; **j** the microscopic structure of oil body in visible light; **k** the microscopic structure of oil body after dyeing by nile red; **l** the statistical chart of oil body partical size. The images were shown at 40 × 10 magnification

Five buffers were compared in the process of oil body extraction. The best extraction rate of 28% was obtained by using PBS buffer (Fig. 1a). The pH value of PBS buffer was screened for pH6 to pH 8.5. The extraction rate was highest, reaching at 31%, at 7.5 (Fig. 1b). When the pH of the oil body suspension was away from the isoelectric point, the electrostatic repulsion increased. When the charge on the droplets increased, the anti-aggregation stability of the droplets rose [29]. The strong electrostatic repulsion prevents from polymerization of oil bodies when pH value was far from the isoelectric point [30].

Following the three-step gradient centrifugation, we extracted the safflower oil body with fewer the purification steps and lower cost. The mature and healthy transgenic seeds with full granules were selected for the oil body extraction. The transgenic seeds were ground in PBS buffer to become suspension (Fig. 1c). The first centrifugation at 12,000 g for 15 min separated the desired upper oil body (Fig. 1d). The oil body was resuspended using PBS buffer of pH 7.5 (Fig. 1e). The oil body suspension liquid was emulsified for 2 min and centrifuged at 1000 g for 15 min separating out the oil bodies (Fig. 1f). The lower liquid was discarded and the upper oil body was cleaned out sequentially (Fig. 1g). A third centrifugation at 8000 g for 15 min purified the oil body (Fig. 1h). Plenty of pure oil bodies were collected as shown in Fig. 1i. The oil bodies were dispersed homogeneously (Fig. 1j) and in good uniformity via nile red under a microscope (Fig. 1k). The diameter of particles from transgenic safflower seeds was mainly in 700–1000 nm (Fig. 1l).

Most of oil body particle size ranged between 1.0 and 1.5 µm in the wild type safflower seeds, however in the transgenic safflower seeds, the diameter of oil body was mostly concentrated in the range of 700–1000 nm. Experimental evidence shows that the size of seed oil bodies is regulated by oleosin [21]. In our approach, oleosin-hEGF-hEGF fusion gene is inserted into the safflower genome, and its expression increases the oleosin content in the surface of oil body, reduces the specific surface area of the transgenic oil body, and reduces the particle size of oil body to the nanometer level. The N-terminus of oleosin was fused with hEGF in transgenic seeds, which increased the quantity of oleosin and then decreased the particle size of oil body. So the transgenic oil bodies became smaller and were conducive to skin absorption. The structure of oil body is similar to the liposome of biological materials, and liposomes can carry drugs and slowly release to the lesion to achieve the therapeutic effect [31]. So the oil body may be used as a new drug delivery system to carry hEGF infiltration into the skin quickly.

### Expression analysis of oleosin-hEGF-hEGF in T3 transgenic safflower

**Fig. 2:**
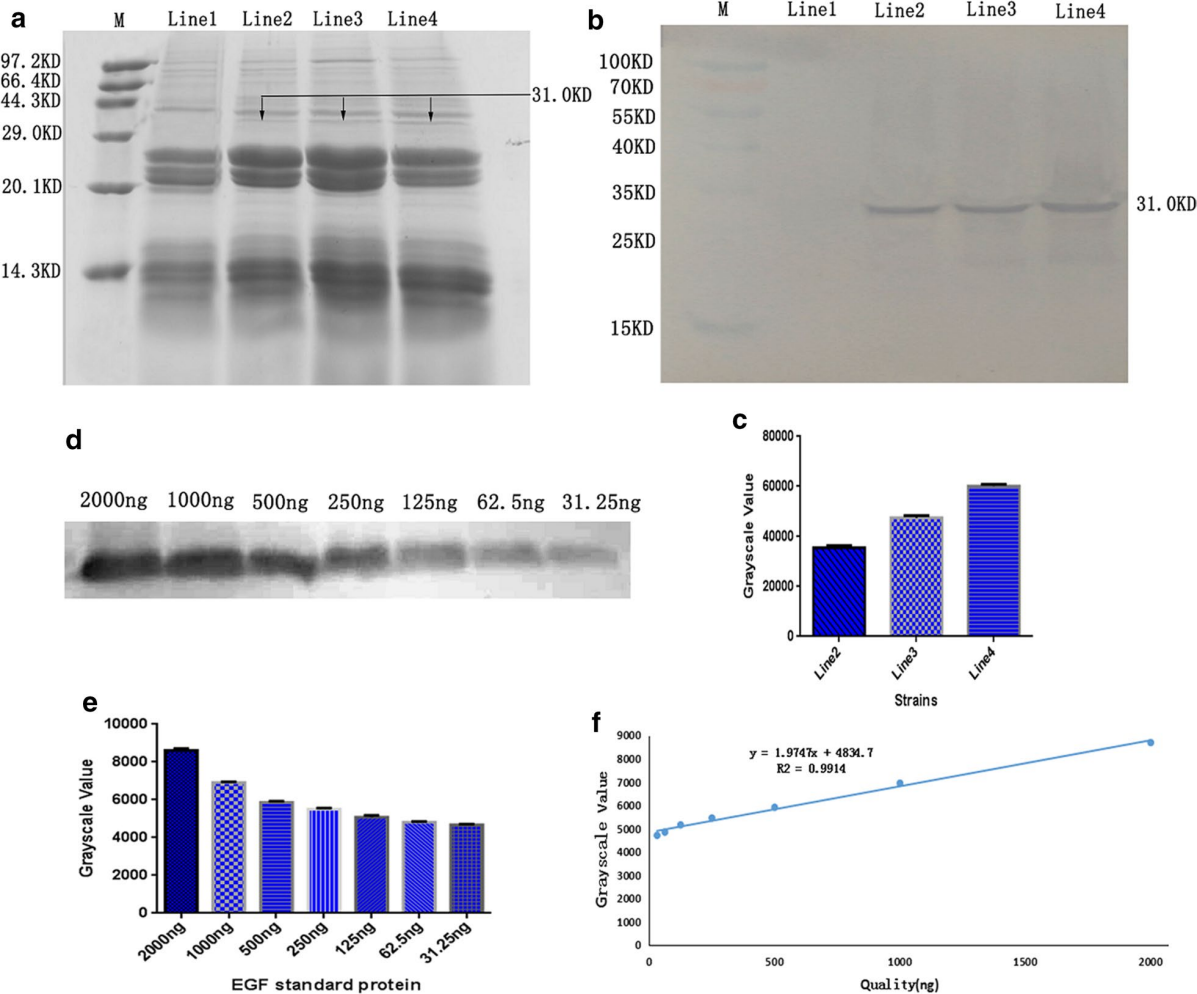
Expression analysis of oleosin-hEGF-hEGF fusion protein in T3 transgenic safflower seeds. **a** Identification of oleosin-hEGF-hEGF from T3 transgenic safflower oil body by SDS-PAGE; **b** detection on oleosin-hEGF-hEGF protein from T3 transgenic safflower oil body by western blotting; **c** oleosin-hEGF-hEGF protein; **d** western blot analysis of different content EGF standard protein; **e** grayscale values analysis of EGF standard protein;** f** the standard curve of the gray values from different content EGF standard protein. *M: protein marker; Line1: the wild-type safflower oil body (WT); Line2–4: the oil body from T3-13, T3-14, T3-17 transgenic seeds

The oil bodies of the three lines from T3 transgenic safflower (T3-13, T3-14, T3-17) were extracted and detected. The hybridization signal was appeared in the oil bodies of three lines. The molecular weight of target band was 31 kDa. However, the wild type oil body did not show hybridization signal (Fig. 2a, b). Thereout, T3-13, T3-14 and T3-17 transgenic lines were positive homozygote but the expression level was different. The expression level of these transgenic lines was calculated through the gray value of the target bands using Quantity One software in Fig. 2c. Meanwhile, the different concentration of EGF standard protein was tested by western blotting (Fig. 2d) and the gray value was analyzed (Fig. 2e). The standard curve was drawn as shown in Fig. 2f. X-axis showed different concentrations of EGF standard protein, Y-axis showed the gray value. When the quantity of EGF standard protein was 2000 ng, the gray value was 8684, and the regression equation was: y = 1.9747x + 4834.79 (Fig. 2f). Finally, the expression level of the target protein was determined according to the gray value of the target band. The expression level from T3-13, T3-14 and T3-17 transgenic seeds were 39.69, 54.88 and 69.32 mg hEGF protein in seeds per gram respectively (Additional file 1: Table S2). The T3-17 transgenic line was selected for subsequent experiments. Yi S et al. [19] expressed fibroblast growth factor 9 and contained 32.9 mg of target protein per gram of transgenic seeds in *Arabidopsis thaliana*. However, our analysis provides evidence that oleosin-hEGF-hEGF is expressed up to 69.32 mg/g in transgenic safflower seeds. This may be due to the expression of a double hEGF gene, which increases the expression of target proteins.

The oil body has wide application, for example, oil body emulsions can be applied in pharmaceutical products, food and feed products and industrial products [18]. Oil body emulsion which has oleosin fused with exogenous protein has been applied to make personal care products [18]. The oil body bioreactors can largely reduce costs. We improved the three-step centrifugation method to reduce the extraction steps and cost. Meanwhile, the expression quantity of oleosin-hEGF-hEGF fusion protein was 69.32 mg/g of safflower seeds. There is no need for purifying the oleosin-bFGF fusion protein, which was fused to oleosin C-terminus and put on the surface of oil bodies to impart its function [19, 32].

### Cell proliferation analysis of oil body expressed oleosin-hEGF-hEGF

**Fig. 3:**
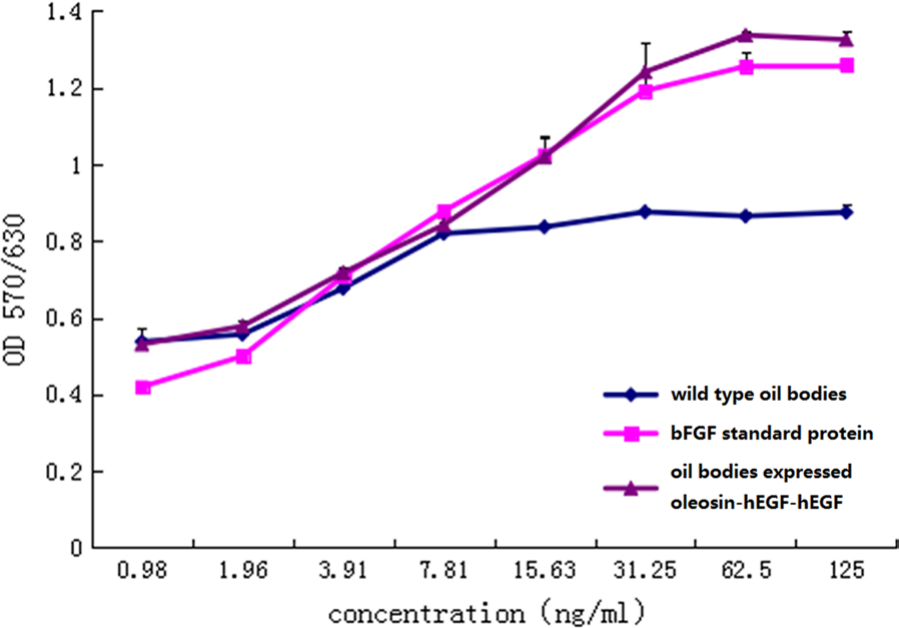
Proliferation activity for the oil body expressed oleosin-hEGF-hEGF on NIH/3T3 cells

Cell proliferation efficiency was detected using the oil body expressed oleosin-hEGF-hEGF fusion protein from T3-17 transgenic seeds. As bFGF protein has the better ability to promote cells proliferation [33], the effectiveness of NIH/3T3 cells proliferation was compared between oil bodies expressed oleosin-hEGF- hEGF fusion protein and bFGF standard protein. The results showed that the oil body expressed oleosin-hEGF-hEGF fusion protein promoted proliferation of NIH/3T3 cells same as bFGF standard protein (Fig. 3). However, the wild type oil body had no noticeable proliferation activity. Thereout, the oil body expressed oleosin-hEGF- hEGF fusion protein from T3-17 transgenic safflower seeds had significant proliferative activity on NIH/3T3 cells.

### Transdermal absorption of the oil body expressed oleosin-hEGF-hEGF

**Fig. 4:**
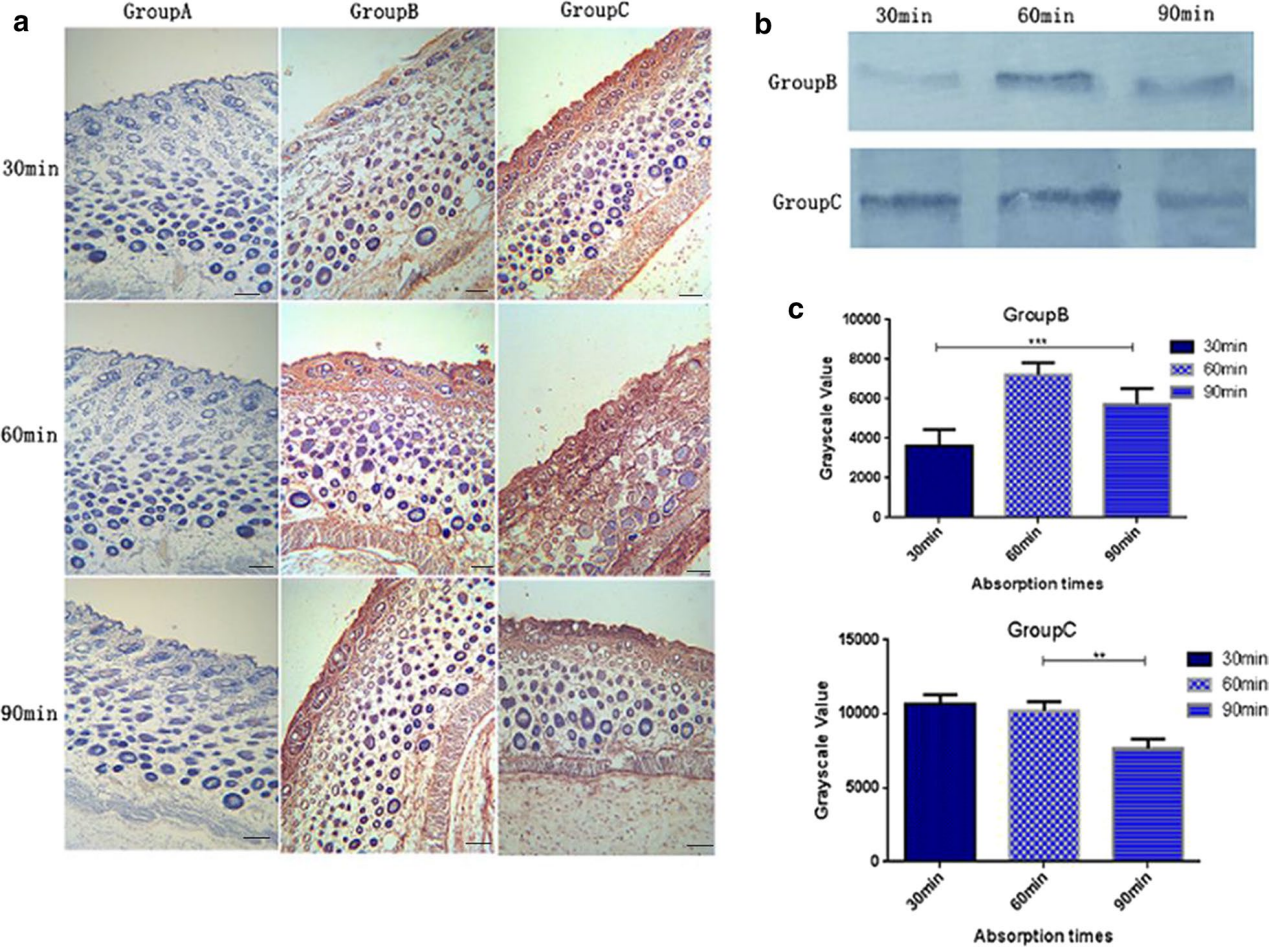
The ability of transdermal absorption from the oil body expressed oleosin-hEGF-hEGF. **a** The evaluation of absorption into skin at different times by immunohistochemistry; **b** western blotting of different times in Group B and Group C; **c** quantification for immunohybridization signal. The images were shown at 40 × 10 magnification

The safflower oil body expressed oleosin-hEGF-hEGF fusion protein was applied to the skin of mice and the ability of penetration into the skin was examined by immunohistochemistry. The positive cells were dyed yellow brown via DAB staining technology. In the group B, the absorption of EGF standard protein was slower than group C after 30 min. The skin surface of the mice still left unabsorbed hEGF protein in group B. The EGF standard protein was mainly concentrated in the epithelial cells and the outer layer of the hair follicles. However, the oil body expressed oleosin-hEGF-hEGF had reached the inner layer of the hair follicles and the epidermal basal layer (Fig. 4a). However, the oil body expressed oleosin-hEGF-hEGF was preferably absorbed in group C and completely absorbed after 60 min (Fig. 4a). This is because these nanosized transgenic oil bodies carrying the hEGF protein are more conducive to skin absorption. At this moment, EGF standard protein remained largely in the outer and epithelial cells of the hair follicles, and a small portion entered the inner layer of the hair follicles. The oleosin-hEGF-hEGF fusion protein was increasingly absorbed, mainly in the inner layer of hair follicles, the epidermal basal layer and the deeper tissues. Most of the proteins in each group were not absorbed from the skin surface after 90 min (Fig. 4a). The expression level of EGF protein in the skin was analyzed (Fig. 4b). The content of EGF protein was maximum at 30 min and retained until 60 min in group C. The gray value was almost the same between 30 and 60 min in group C (Fig. 4c). However, the content of EGF protein reached a maximum at 60 min in group B (Fig. 4c). These data demonstrate that oil body carrying oleosin-hEGF-hEGF can penetrate rapidly into the skin tissue and the amount of absorption is higher than the EGF standard protein group.

The structure of oil body is similar to liposome [34]. The liposome can carry drugs into the wound tissue [35]. The penetration mechanisms of oil body may be similar to lipsome. Liposome-carried drugs can promote the internalization of the loaded drugs into the cells [36]. After a certain period of interaction, the drug aggregates around the nuclear membrane and after a certain period of time, the liposome-carried the drug penetrates into the epidermis, and the liposomes exhibit a strong transdermal penetration capability. The oil body carried hEGF infiltrates into the dermis and promotes the penetration of the drug into the dermis. The oil body has a similar structure of liposome, so we speculated that the oil body carried hEGF penetrates into the skin with the same mechanism and achieves better absorption effect.

### Oil body expressed oleosin-hEGF-hEGF accelerates wound healing in rats

**Fig. 5:**
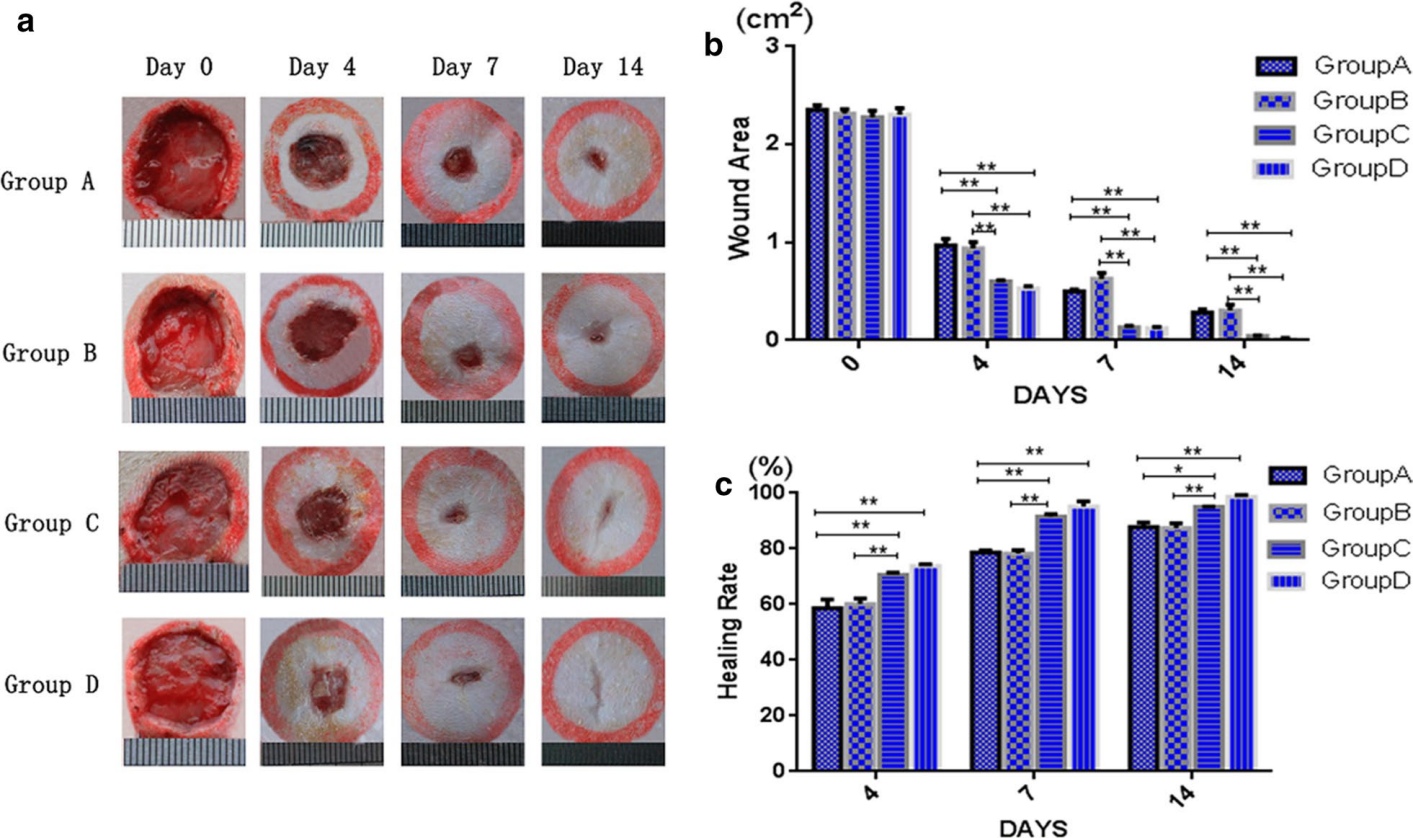
The oil body expressed oleosin-hEGF-hEGF accelerated wound closure. **a** The photograghs of wound closure after four treatment group at day 0, 4, 7, and 14; **b** wound area of four groups at day 0, 4, 7, and 14; **c** wound healing rates of four groups at day 4, 7, and 14. *Group A: normal saline; Group B: wild type oil body; Group C: EGF standard protein; Group D: Oil body expressed oleosin-hEGF-hEGF

To evaluate the efficacy of oil body expressed oleosin-hEGF-hEGF in accelerating cutaneous wound healing, full-thickness cutaneous wounds in wistar rats were tested. The photographs of wound in four treatment groups on day 0, 4, 7 and 14 were taken. The wounds of four treatment groups were coalesced gradually during 14 days. The wound of the treatment group D showed significantly faster closure than the other treatment groups (Fig. 5a). The wound areas of group C and group D were decreased more than group A and group B on day 7 and day 14 as shown in Fig. 5b. The wound healing rate of group C and group D exceeded 90% after treatment for 7 days, while in the same duration, wound healing rate was less than 80% in the group A and group B (Fig. 5c). The surfaces of wounds from group D were almost healed and appeared almost scarless after 14 days of treatment, whereas the wound healing of group C was near completion. On the hand, the wounds in group A and the group B were healed incompletely, as shown in Fig. 5c. The wound closure rate in group D was higher than the other groups at all treatment times (day 4, 7 and 14). Thus, the oil body expressed oleosin-hEGF-hEGF fusion protein in safflower seeds played an important role in skin wound healing.

At present, hEGF has been developed in different dosage form in skin wound healing research. For example, Hori et al. [37] prepared hEGF as a cationic gelatin hydrogel to promote corneal epithelial wound healing by controlled release. Choi et al. [38] combined hEGF with protamine of low-molecular weight for skin wound healing. Goh et al. [39] loaded hEGF with heparin-based hydrogel sheet for skin wound healing. In addition, the oil body expressed oleosin-hEGF- hEGF could repair the wound significantly.

### Oil body expressed oleosin-hEGF-hEGF improves granulation formation

**Fig. 6:**
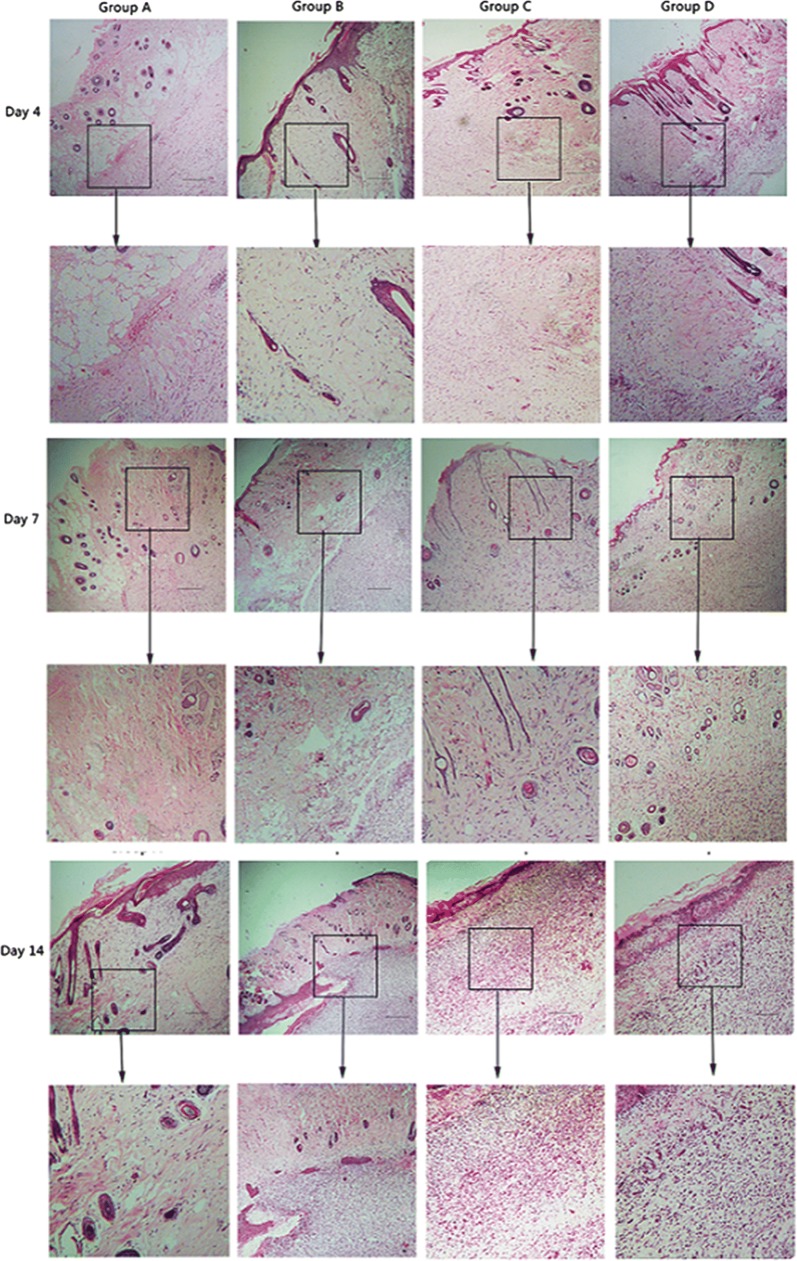
Hematoxylin and Eosin images for different groups in wound healing. *Group A: normal saline; Group B: wild type oil body; Group C: EGF standard protein; Group D: oil body expressed oleosin-hEGF-hEGF. The images were shown at 40 × 10 magnification (Scale bars = 100 µm)

The wound healing is mainly accomplished by the proliferation of granulation tissue and lateral migration of epidermal cells. The histological observation of H&E is consistent with the wound healing rate. The wound tissues of four treatment groups were stained with H&E on day 4, 7 and 14. The wounds had healed incompletely in group A and group B, but the skin structure was regenerated completely in group C and group D. On the other hand, the granulation tissue had provided a temporary matrix with cells to repair the wound. Histological detection of granulation thickness was followed as shown in Fig. 6. H&E showed the regeneration of epidermis cells and granulation quantity. The quantity of granulation from group D augmented increasingly until day 14 and it closely arranged in day 14. The quantity of granulation tissue in group D was more than others. The number of granulation tissue was getting thicker and arranged more regular in group D. It was shown that oleosin-hEGF-hEGF could promote the regeneration of epidermal cells during wound healing, improved the formation of granulation and the compactness of tissue arrangement, and which had a good effect of promoting wound healing.

### Oil body expressed oleosin-hEGF-hEGF facilitates collagen deposition

**Fig. 7:**
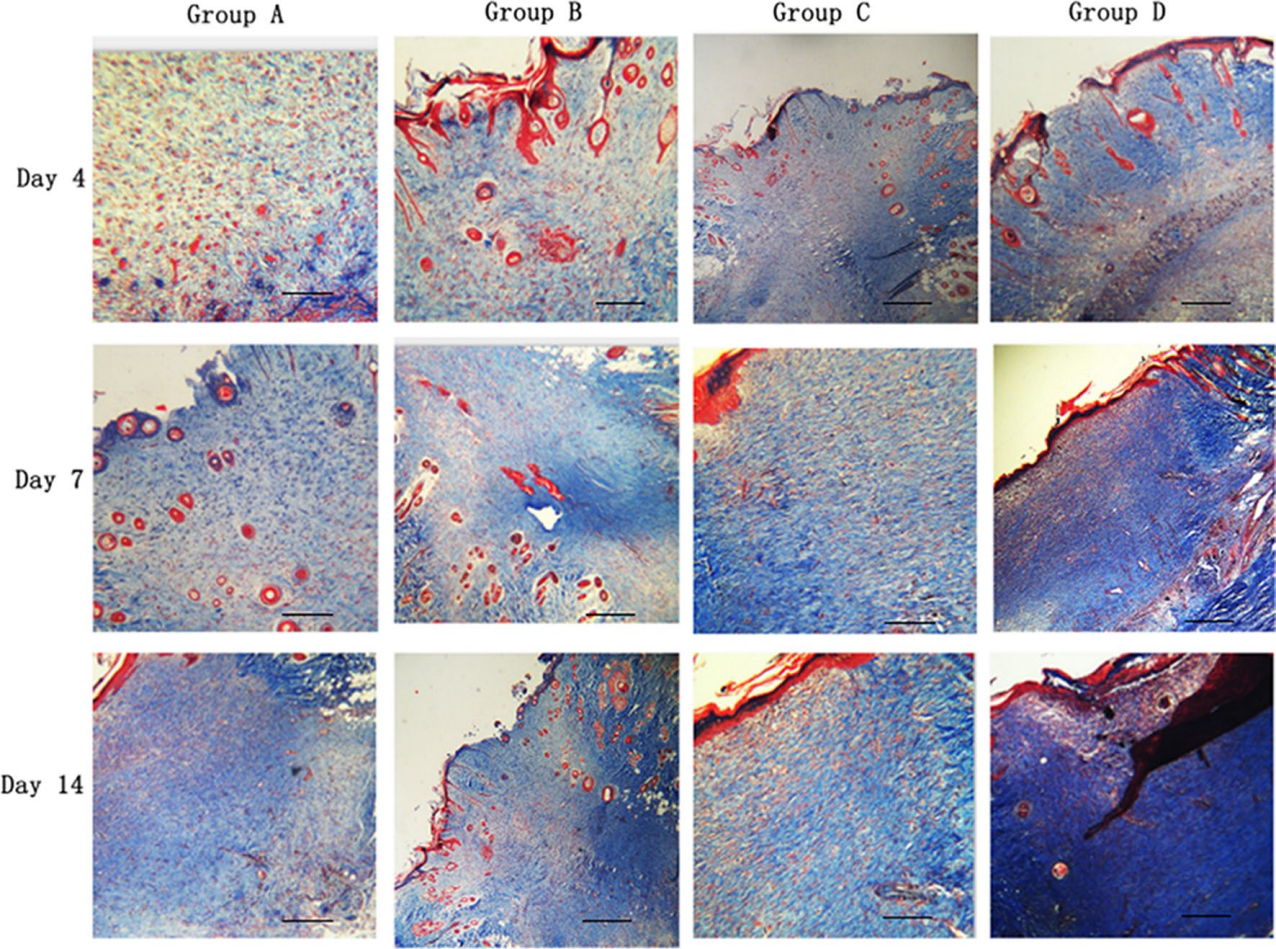
Histochemical staining of collagens deposition in process of wound healing. Tissue sections are stained by Masson’s trichrome (blue: collagen; red: cytoplasm and muscle fibers). The images were shown at 40 × 10 magnification (scale bars = 100 µm). *Group A: normal saline; Group B: wild type oil body; Group C: EGF standard protein; Group D: oil body expressed oleosin-hEGF-hEGF

The deposition of collagen determines the size and appearance of newborn skin scars [28, 40–42]. The oil body expressed oleosin-hEGF-hEGF could promote collagen deposition during the wound repairing in four groups and the amount of collagen was produced increasingly by masson staining at day 4, 7 and 14 (Fig. 7). On day 4, four groups behaved different measures of collagen deposition, but it was not obvious in group A. The synthesis of collagen protein in the group D was greatly increased in day 7, even higher than other groups. The control group A also had a certain effect to promote collagen synthesis, but they had the least accumulation of collagen (Fig. 7). The collagen deposition appeared among the four groups and the oil body-treated wounds had the highest ranked collagen fibers at day 14. In the same treatment time, the deposition of collagen was also changed in four groups. But the deposition of collagen was maximized in group D at day 14. These results showed that oleosin- hEGF-hEGF fusion protein expressed in safflower oil body can effectively promote the skin wound collagen deposition to facilitate wound healing.

### Oil body expressed oleosin-hEGF-hEGF induced cell proliferation activity

**Fig. 8:**
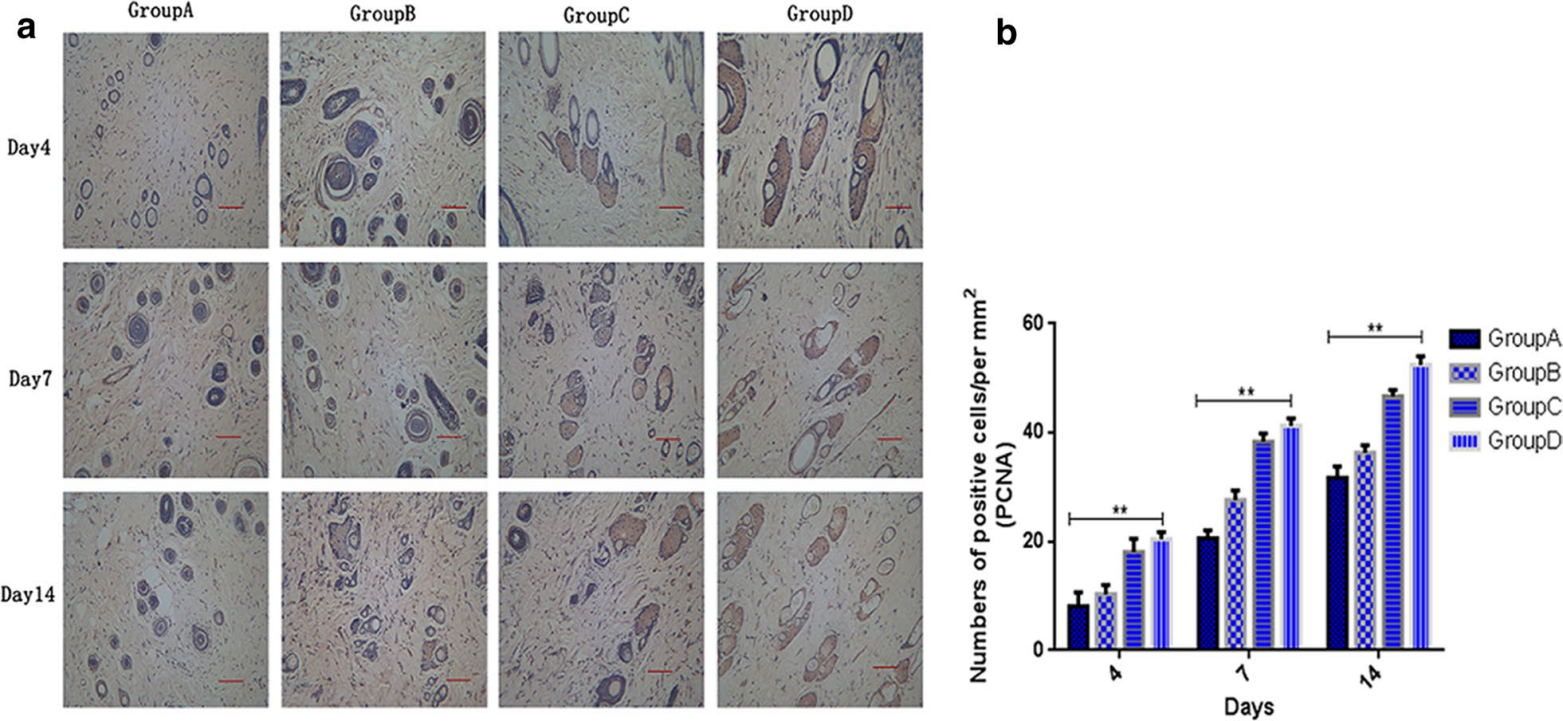
The formation of keratinocytes and quantitative analysis. **a** PCNA immunohistochemistry staining on different days in mice, showing enhanced cellular proliferation. The images were shown at 20 × 10 magnification (scale bar = 50 μm). **b** Quantitative analysis of relative density of PCNA at day 4, 7, 14 after surgery. *Group A: normal saline; Group B: wild type oil body; Group C: EGF standard protein; Group D: oil body expressed oleosin-hEGF-hEGF

We studied tissue immunohistochemistry to observe the cell proliferation activity by measuring the expression of a labeled nuclear antigen (Proliferating Cell Nuclear Antigen, PCNA). The oleosin-hEGF-hEGF treatment group showed increased number of PCNA-positive cells in the wound on the 7th day than the other groups (Fig. 8). It indicated the cell proliferative activity of oleosin-hEGF-hEGF. In addition, it was able to promote the expression of PCNA in epithelial and dermal tissues simultaneously. It was also confirmed that oleosin-hEGF-hEGF could enhance the expression level of PCNA in tissues, which is also consistent with H&E, Masson, and the results of the above data. Therefore, this experiment also confirmed that oil body expressed oleosin-hEGF-hEGF can stimulate cell proliferation and secretion of large amounts of ECM substrate promoting the healing of skin wound.

### Oil body expressed oleosin-hEGF-hEGF increases vascularization

**Fig. 9:**
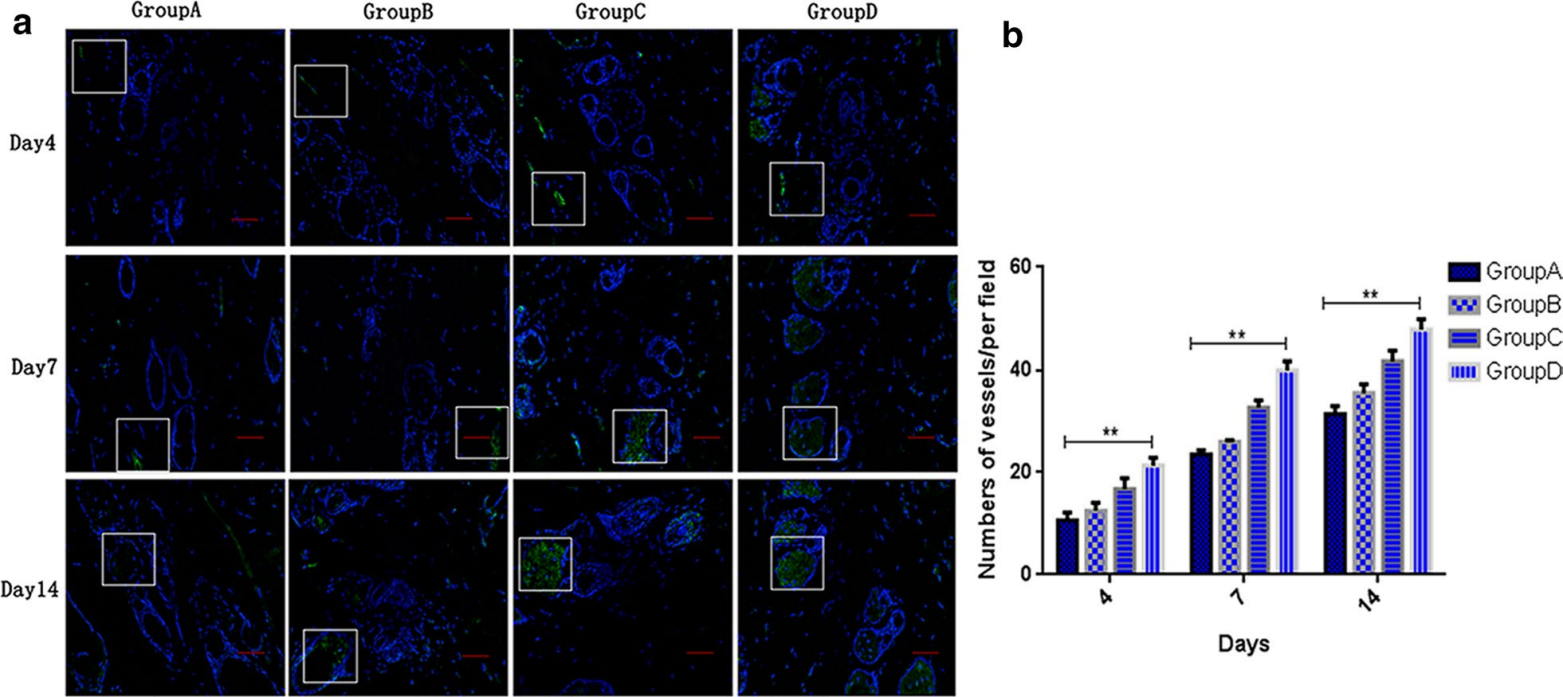
The formation of new blood vessels and quantitative analysis. **a** New blood vessels stained with CD31 (green) and DAPI (blue) at day 4, 7, 14 after surgery. The images were shown at 20 × 10 magnification (scale bar = 50 μm); **b** quantitative analysis of number of vessels per field at day 4, 7, 14 after surgery. *Group A: normal saline; Group B: wild type oil body; Group C: EGF standard protein; Group D: oil body expressed oleosin-hEGF-hEGF

During the skin wound repairing, it is essential that the new blood vessels are formed. To assess neovascularization, we used CD31 as an endothelial cell marker and calculated its expression and immunized the CD31 in the blood vessels with skin sections to stain them green and DAPI was then dyed to make the nucleus blue. In Fig. 9a, the immunofluorescence assay of CD31 in each treatment group confirmed the number of neovascularization for each time. The largest number of neovascularization was observed in the oleosin-hEGF-hEGF treatment group, which was slightly higher than that of the EGF protein group, and only a small amount of neovascularization was observed in the normal saline group. In Fig. 9b, the vascular density in the wounds of each treatment group was compared and it was higher in the oleosin-hEGF-hEGF treatment group than other groups. These data indicated that oil body expressed oleosin-hEGF-hEGF can effectively accelerate skin wound repair by stimulating the vascular growth of the wound.

### The change of endogenous TGF-β1 factor after treatment

**Fig. 10:**
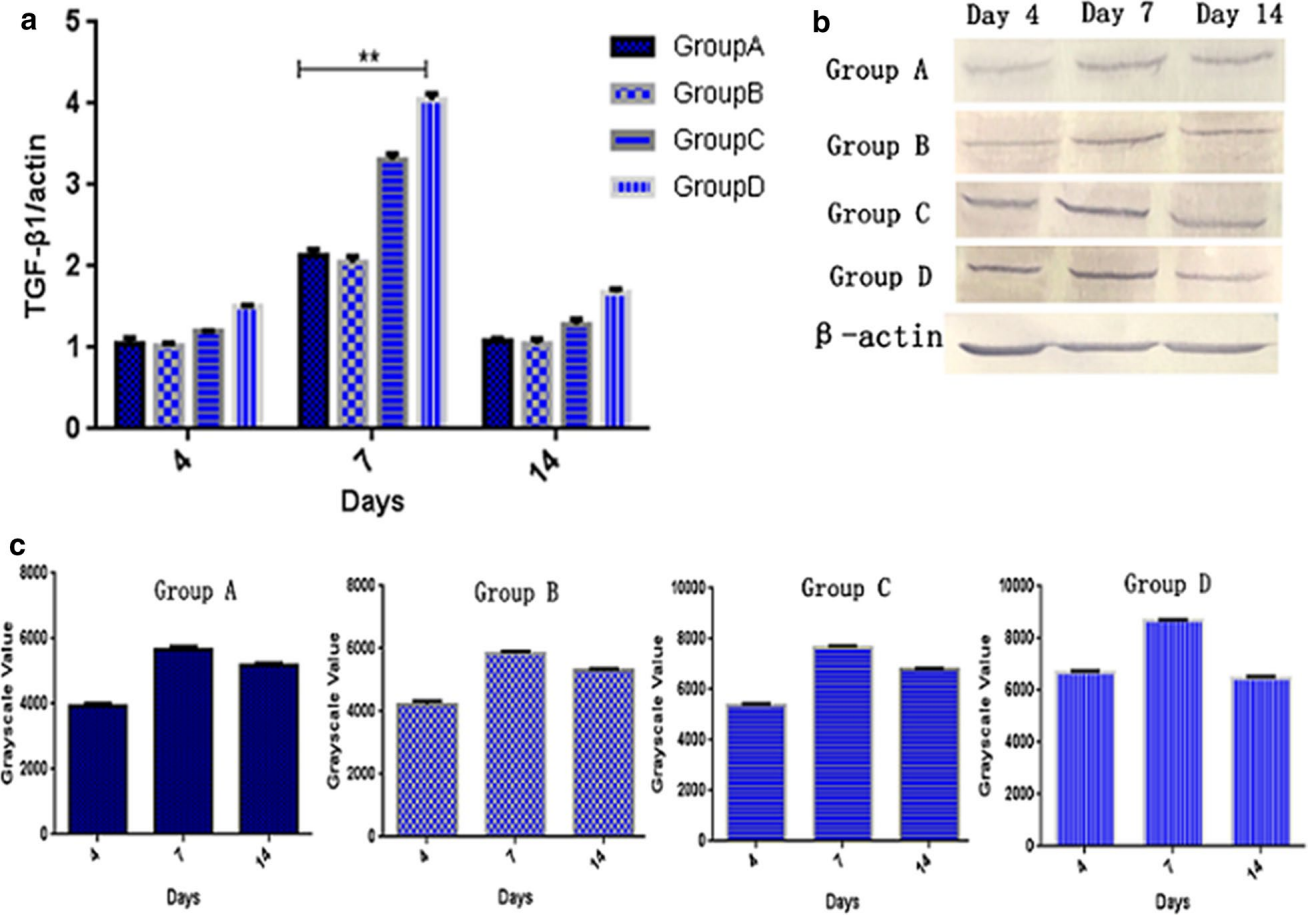
Transcription and expression assay of endogenous TGF-β1 in process of skin wounds healing. **a** RT-qPCR detection on endogenous TGF-β1 mRNA of four groups at day 4, 7, and 14; **b** expression analysis of endogenous TGF-β1 protein of four groups at day 4, 7, and 14 by western blotting; **c** quantification for immunohybridization signal by grayscale values. *Group A: normal saline; Group B: wild type oil body; Group C: EGF standard protein; Group D: oil body expressed oleosin-hEGF-hEGF

There are a number of factors involved during the skin wound repairing. The proliferation and migration of cells are mainly regulated by bFGF [43–45], the formation of collagen in the skin is mainly affected by TGF [46] and the regeneration of skin blood vessels is mainly affected by VEGF [32]. For TGF-β1 factor, it can be seen that the transcriptional levels of four groups had no difference on day 4, but the oil body expressed oleosin-hEGF-hEGF group was highest as shown in Fig. 10a. On day 7, the TGF-β1 transcriptional levels of each group were significantly increased, especially oil body expressed oleosin-hEGF-hEGF group. On the 14th day, the TGF-β1 transcriptional levels of four groups decreased to the normal level, but the transcriptional level of oil body expressed olesoin-hEGF group was still relatively high (Fig. 10a). In accordance with analysis of the TGF-β1 protein, the level of the expression of TGF-β1 in the treatment group of oil body expressed oleosin-hEGF-hEGF was significantly higher than other groups (Fig. 10b, c). The level of the expression of TGF-β1 gradually increased on day 4 to day 7, but that of TGF-β1 was significantly decreased on the 14th day, as shown in Fig. 10b, c.

### The effect of oil body expressed oleosin-hEGF-hEGF on endogenous bFGF factor

**Fig. 11:**
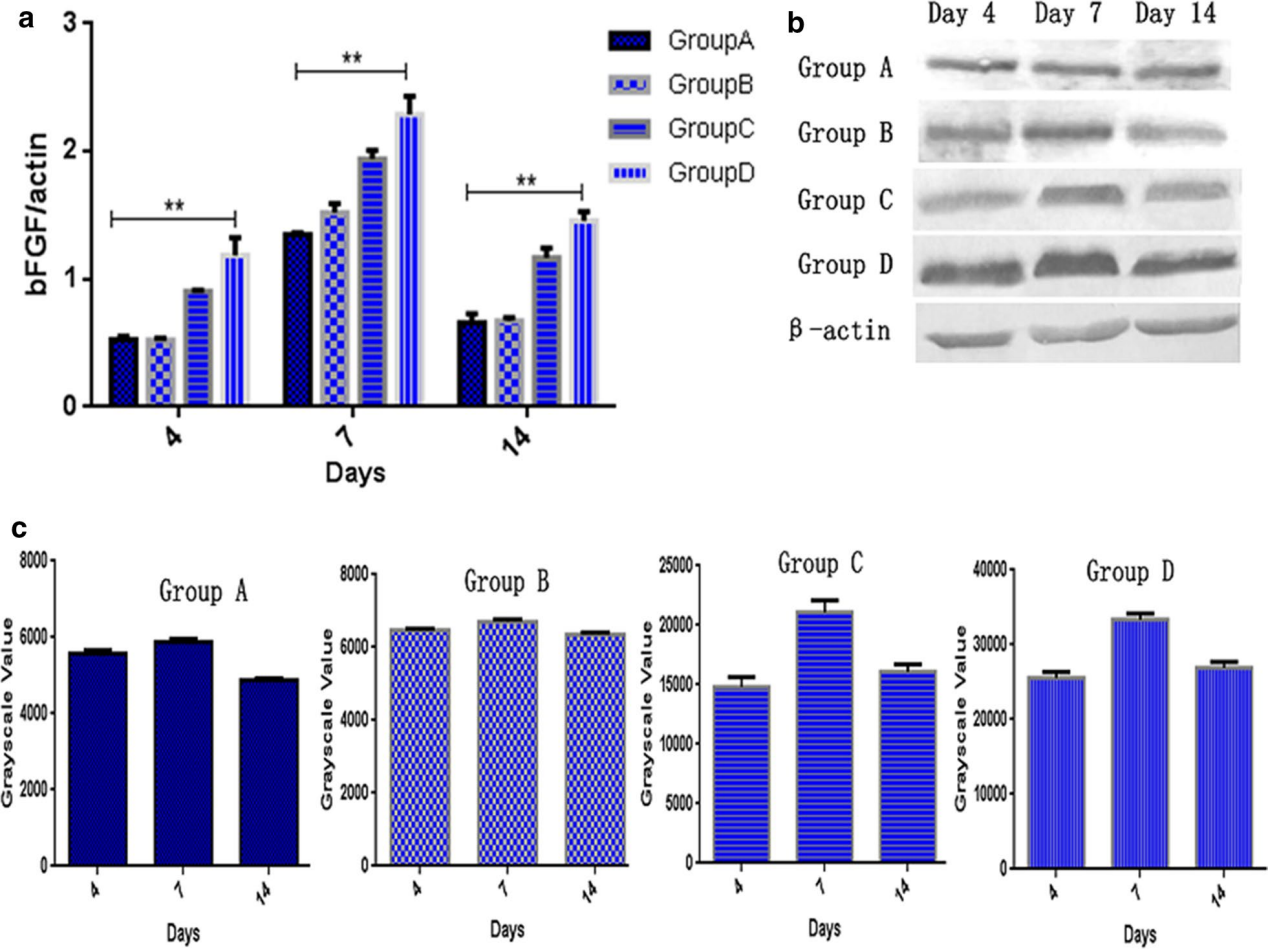
Transcription and expression assay of endogenous bFGF in process of skin wounds healing. **a** RT-qPCR detection on endogenous bFGF mRNA of four groups at day 4, 7, and 14; **b** expression analysis of endogenous bFGF protein of four groups at day 4, 7, and 14 by western blotting; **c** quantification for immunohybridization signal by grayscale values. *Group A: normal saline; Group B: wild type oil body; Group C: EGF standard protein; Group D: oil body expressed oleosin-hEGF-hEGF

At the same time, we also detected the transcriptional level of endogenous factor bFGF (Fig. 11). For the endogenous factor bFGF involved in skin wound healing, the transcriptional level of bFGF in the oil body expressed oleosin-hEGF-hEGF group was higher than others on the 4th day (Fig. 11a). On the 7th day, the transcriptional level of bFGF was significantly increased in each group, and the bFGF transcriptional level of oil body expressed oleosin-hEGF-hEGF treatment group was the highest. On the 14th day, the transcriptional level of bFGF was significantly decreased in each group, but the bFGF transcriptional level was higher than the normal level (Fig. 11a). In accordance with the bFGF protein expression, the expression of bFGF in the group of oil body expressed oleosin-hEGF-hEGF was much higher than others. In addition, the expression of bFGF gradually increased in each group on day 4 to day 7. However, the expression of bFGF in the normal saline group and the wild-type oil body group was not notably elevated as shown in Fig. 11b, c. The bFGF expression was significantly decreased on the 14th day (Fig. 11b, c).

### The effect of oil body expressed oleosin-hEGF-hEGF on endogenous VEGF factor

**Fig. 12:**
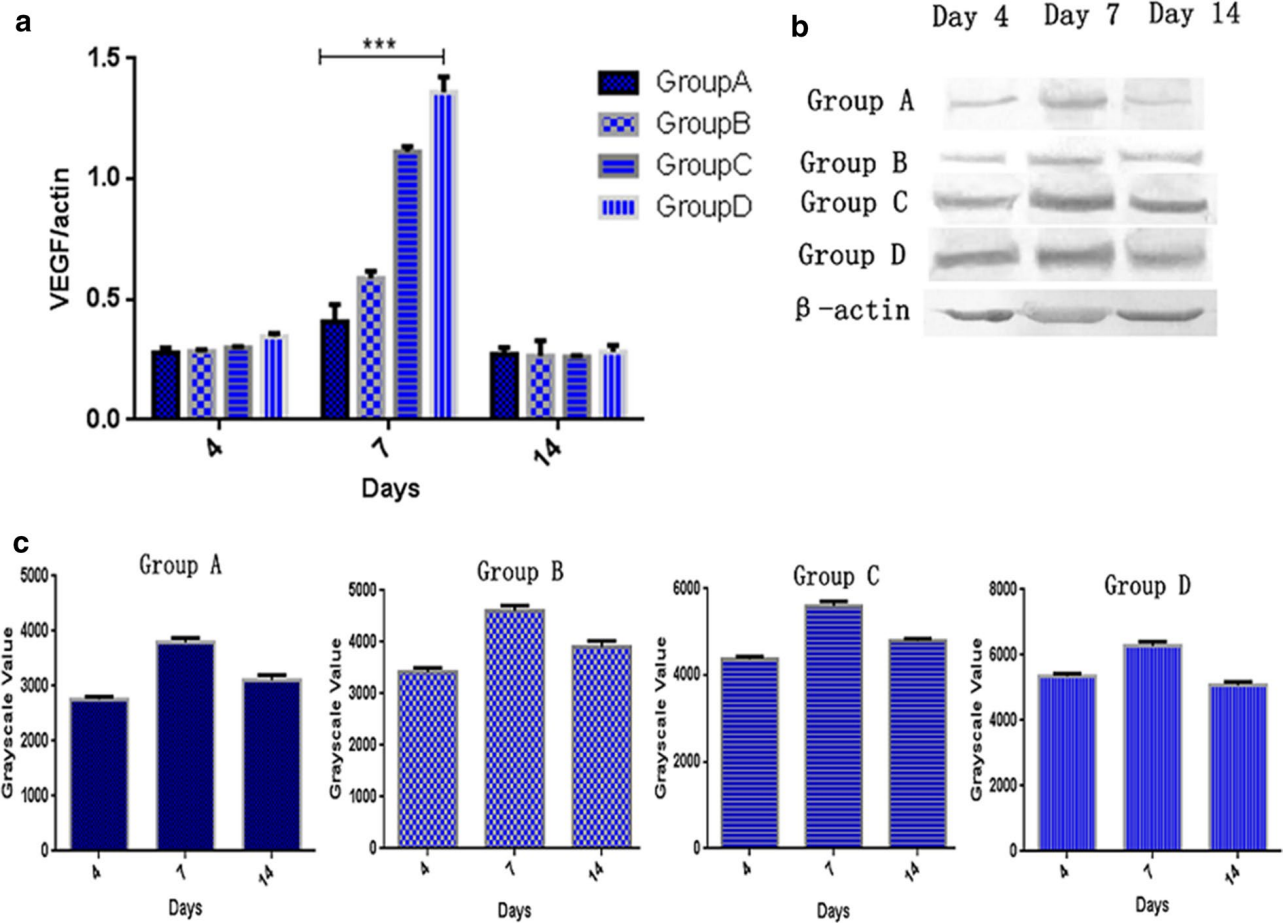
Transcription and expression assay of endogenous VEGF in process of skin wounds healing. **a** RT-qPCR detection on endogenous VEGF mRNA of four groups at day 4, 7, and 14; **b** expression analysis of endogenous VEGF protein of four groups at day 4, 7, and 14 by western blotting; **c** quantification for immunohybridization signal by grayscale values. *Group A: normal saline; Group B: wild type oil body; Group C: EGF standard protein; Group D: oil body expressed oleosin-hEGF-hEGF

Meanwhile, the expression of endogenous factor VEGF, which controls the blood vessels in the process of skin regeneration, was also detected by transcriptional level and protein expression analysis as shown in Fig. 12. On the 4th day, the transcriptional levels of VEGF in each group were similar, but slightly higher in the oil body expressed oleosin-hEGF-hEGF group under control of angiogenic factor VEGF in the healing process of skin wound (Fig. 12a). On the 7th day, the transcriptional levels of VEGF in the oil body expressed oleosin-hEGF-hEGF group and EGF standard protein group were significantly increased, however, VEGF in the oil body expressed oleosin-hEGF-hEGF group was a bit higher than VEGF content in the EGF standard protein group. The expression in wild-type oil body group and the normal saline group were also increased slightly (Fig. 12a). On the 14th day, the transcriptional level of VEGF in each group decreased significantly until the normal level (Fig. 12a). The expression of VEGF in the treatment group of oil body expressed oleosin-hEGF-hEGF was much higher than the expression of VEGF in normal saline group and EGF standard protein group. The expression of VEGF gradually increased in each treatment group on day 4 to day 7, but the expression level of VEGF was significantly decreased on the 14th day as shown in Fig. 12b, c.

The oil body containing oleoin-hEGF-hEGF was very effective on wound healing, and its effect was slightly superior to EGF protein. This may be because the oil body contains a lot of unsaturated fatty acids and vitamin E, which may positively serve as in repair of skin trauma [47]. At the same time, we detected the transcription and expression of TGF-β1, bFGF and VEGF in wound repair mechanism, and found that the expression level of these factors displayed a trend of rise first then fall during the wound repairing. It is probably because the skin wound was initially in the inflammatory period and achieved the proliferative period within 7 days and thus the content of these factors is increased in this period to accelerate wound healing. After oleoin-hEGF-hEGF smeared on the wounds of the skin, it activates EGFR which promotes the production of TGF-β1 [48]. In normal skin tissue, the expression of TGF-β1 is very low. After the skin is traumatized, TGF-β1 is released by platelets and epidermal cells [37]. TGF-β1 as a chemokine is capable of recruiting bFGF and VEGF to wound sites [49, 50]. Due to traumatic epidermis damage, blood exudation and other reasons, TGF-β1, bFGF and VEGF level is not high during a few hours, but in the inflammatory phase, TGF-β1 began to induce large-scale production of bFGF and VEGF [49]. Therefore, the expression levels of these three factors increase rapidly in the beginning and maintain at a high level, but when the wound is healed, they return to normal level again. At this time, VEGF helps prolife rate vascular endothelial cells and the formation of neonatal capillary [51]. TGF-β1 can induce the accumulation of neutrophils and macrophages to the wound, promote proliferation of fibroblast and cell matrix synthesis [52]. bFGF is mainly involved in the synthesis of collagen fibers [53].

## Conclusion

Safflower oil body was used as a bioreactor to obtain oleosin-hEGF-hEGF protein. A large number of transgenic safflower oil bodies were obtained at high yield. The extraction method of safflower oil body was optimized. The pure oil body was obtained from the T3 transgenic safflower seeds. The diameter of oil body was mostly concentrated in the range of 700–1000 nm in the transgenic safflower seed. The particle size was reduced significantly in transgenic seeds and it was extremely beneficial to skin absorption. The expression level of oleosin-hEGF-hEGF in safflower seeds was 80.43 ng/μL oil body. The oil body expressed oleosin-hEGF-hEGF had significant proliferative activity on NIH/3T3 cells and improved skin regeneration to accelerate wound healing in rats. It was found that the neonatal fibroblasts and collagen was increased in the safflower oil body expressed oleosin-hEGF-hEGF treatment group by H&E and Masson. Finally, the oil body expressed oleosin-hEGF-hEGF could accelerate wound healing rate and stimulate expression of related growth factors during skin tissue repair. The dynamic expression levels of TGF-β1, bFGF, VEGF were increased after treatment 4 days and 7 days, but decreased after 14 days. Therefore, it can availably promote skin regeneration to accelerate wounds healing.

## Additional file

**Additional file 1: Table S1.** Primer sequence. Table S2. The expression level of hEGF in T3 transgenic seeds of safflower.

## Abbreviations

hEGF: human epidermal growth factor
EGFR: epidermal growth factor receptor
PVDF: polyvinylidene fluorid
MTT: 3-(4,5-dimethyl-2-thiazolyl)-2,5-diphenyl-2-H-tetrazolium bromide
DMEM: dulbecco’s modified eagle media
DMSO: dimethyl sulfoxide
PCNA: proliferating cell nuclear antigen
ECM: extracellular matrix
CD31: platelet endothelial cell adhesion molecule-1
DAPI: 4′,6-diamidino-2-phenylindole
HE: hematoxylin-eosin
TGF-β1: transforming growth factor beta 1
bFGF: basic fibroblast growth factor
VEGF: vascular endothelial growth factor
