# Supplementary material for: A new nanoscale transdermal drug delivery system: oil body-linked oleosin-hEGF improves skin regeneration to accelerate wound healing
Source: J Nanobiotechnology. 2018 Aug 30;16:62. doi: 10.1186/s12951-018-0387-5 (PMC6116364; doi:10.1186/s12951-018-0387-5)
Supplement: Supplementary file 1 — Additional file 1: Table S1. Primer sequence. Table S2. The expression level of hEGF in T3 transgenic seeds of safflower. [file 12951_2018_387_MOESM1_ESM.doc]

Additional file

**A new nanoscale transdermal** **drug delivery system: oil body-linked oleosin-hEGF improves** [**skin regeneration**](javascript:;) **to accelerate wound healing**

Weidong Qiang^1#^, Tingting Zhou^2#^, Xinxin Lan^1#^, Xiaomei Zhang^1^, Yongxin Guo^1^, Muhammad Noman^1^, Linna Du^1^, Jie Zheng^1^, Wenqing Li^1^, Haoyang Li^1^, Yubin Lu^1^, Hongyu Wang^1^, Lili Guan^1^, Linbo Zhang^1^, Xiaokun Li^1*^, Jing Yang^1*^, Haiyan Li^1*^

^1^ College of Life Science, Engineering Research Center of the Chinese Ministry of Education for Bioreactor and Pharmaceutical Development, Jilin Agricultural University, Changchun130118, China

^2^ Jilin KingMed Center for Clinical Laboratory Co.,Ltd, Changchun130000, China

# The authors contributed equally to this work.

*Corresponding Authors:

Jing Yang, E-mail:yangjing5122010@163.com;

Haiyan Li, E-mail: [hyli99@163.com;](mailto:hyli99@163.com(H.Y.Li);)

Xiaokun Li, E-mail : xiaokunli@163.net

**Experimental Section**

TableS1. Real-time quantitative reverse transcription PCR (RT-qPCR) was performed by SYBR Premix Ex Taq™ kit (Takara, Japan) on Stratagene Mx3000P thermocycler (Agilent). Gene-specific primers for the TGF-β1, bFGF, VEGF genes and candidate reference β-actin gene were designed.

TableS2. The oleosin-hEGF fusion protein accumulation data in safflower seeds was [analyzed](app:ds:analyze) with Quantity One [software](app:ds:software).The expression level of the target protein was determined according to the gray value of the target band. The expression level from T3-13, T3-14 and T3-17 transgenic seeds were 39.69mg, 54.88mg and 69.32mg hEGF protein in seeds per gram respectively (Tab.S2).

**Tab.**S1. Primer Sequence

| **Primer Name** | **Primer Sequence** |
| --- | --- |
| β-actin | Forward：5’-CCTCTATGCCAACACAGTGC-3’ |
|  | Reward：5’-GTACTCCTGCTTGCTGATCC-3’ |
| TGF-β1 | Forward: 5’-GAGGCGGTGCTCGCTTTGT-3’ |
|  | Reward：5’-TGTTGCGGTCCACCATTAGC-3’ |
| VEGF | Forward：5’-CCAGGAGTACCCCGATGAGATAG-3’ |
|  | Reward：5’-CTGGCTTTGGTGAGGTTTGATC-3’ |
| bFGF | Forward：5’-AAGCAGAAGAGAGGAGTTG-3’ |
|  | Reward：5’-CGGTAAGTGTTGTAGTTATTGG-3’ |

**Tab. S2. The expression level of hEGF in T3** transgenic seeds of safflower

|  | **Grayscale value** | **The quality of hEGF per microlitre oil body (ng)** | **The quality of hEGF per gram seeds (μg)** |
| --- | --- | --- | --- |
| **T3-13** | 36186.22 | 1984.57 | 39691.47 |
| **T3-14** | 48183.90 | 2744.03 | 54880.74 |
| **T3-17** | 59590.61 | 3466.09 | 69321.80 |
